# Interparticle and Brownian forces controlling particle aggregation and rheology of silicate melts containing platinum-group element particles

**DOI:** 10.1038/s41598-022-12948-1

**Published:** 2022-06-02

**Authors:** Luiz Pereira, Jérémie Vasseur, Fabian B. Wadsworth, Frank Trixler, Donald B. Dingwell

**Affiliations:** 1 Department of Earth and Environmental Sciences, Ludwig-Maximilians-Universität München, 80333 Munich, Germany; 2 Department of Earth Sciences, Durham University, Durham, DH1 3LE UK; 3 Center for NanoScience (CeNS), Ludwig-Maximilians-Universität München, 80799 Munich, Germany

**Keywords:** Environmental sciences, Engineering, Materials science

## Abstract

We study the rheology of silicate melts containing platinum-group element (PGE) particles. They exhibit a shear-thinning behaviour, an intense aggregation tendency, and an anomalously high apparent viscosity in the low shear rate limit, even at very low particle volume fraction. Using a compilation of published experimental data, we analyse these effects in three steps. Firstly, we observe that the viscosities of these suspensions are much higher than those of natural silicate crystal-bearing melts for low shear rate regimes. Secondly, we demonstrate that the viscosities at low shear rate limit cannot be estimated by classical rheological models but rather may be understood as the result of particle aggregation, trapping dead fluid, and thereby increasing the effective particle volume fraction. Finally, we scale the critical shear rates for shear-thinning using a Peclet number analysis—invoking a competition between random thermal particle motion and hydrodynamic shearing motion—and, using an empirical extension, we additionally account for the particle–particle interaction energetics. We propose a framework in which the rheology of this family of particle-bearing melts can be predicted, and demonstrate that at low Peclet numbers, PGE-bearing particle aggregation is driven by interparticle forces and Brownian motion.

## Introduction

The presence of crystalline solids suspended in melts affects many properties of silicate systems, such as electrical conductivity, dynamics of bubbles, onset of viscoelasticity, and apparent suspension viscosity^1–9^. Partially crystallised silicates with moderate-to-high crystal volume fractions ($\phi > 0.2$) can exhibit apparent viscosities many orders of magnitude higher than that of the pure liquid phase^10–14^. Notably, experiments show that in some cases, at low rates of shear, even a comparatively small volume fraction ($\phi$ ~ 0.02) of particles can increase the system viscosity by the same amount. This effect has been observed for suspensions where the inclusion phase contains PGEs, such as RuO_2_ crystals, implying that these PGE-bearing particle types have an effect distinct from that of other crystal types^15–17^. It has been proposed that these PGE-bearing particles suspended in silicate melts increase the system viscosity anomalously due to their tendency to aggregate by interparticle forces^15–17^. Yet the detailed mechanisms controlling this effect, and predictive models for this behaviour, remain unclear. This is despite the fact that aggregation of suspended particles in fluids is of increasing interest in fields for which active nano- to microscopic particles are present in liquids, including polymer science, food science, and medicine^18–20^. Under flow, these so-called colloidal particles may experience a range of forces (*i.e.* hydrodynamic, Brownian, and interparticle forces), which in turn determine their internal structure and consequently the rheological response of the system to applied stress.

In addition to the anomalously high apparent viscosity observed for these suspensions at low shear rates, a shear-thinning rheological response is observed, which connects a high viscosity plateau at low shear rates with a low plateau viscosity at high shear rates^15–17^. The critical shear rate for shear-thinning remains poorly predictable for these systems, a likely consequence of the lack of a full understanding of the mechanisms at play during flow.

To understand better the rheological behaviour of silicate melts with PGE-bearing particles, we collated existing experimental data from published sources^15–17^. Here, we present a brief description of the extracted data as well as the rheological behaviour of such systems, then demonstrate that PGE-bearing particles can increase the viscosity of silicate melts much more than can silicate crystals. We confirm that classical rheological models cannot estimate well the viscosity behaviour of melts with PGE-bearing particles at low shear rate without accounting for effective changes in the particle volume fraction due to aggregation. We apply then a scaling approach to the collated literature data and obtain a universal description of the shear-thinning behaviour. Finally, we explore the extent of supporting evidence for aggregation and an interparticle force in suspensions of PGE-bearing particles.

## Rheology of silicate melts containing PGE-bearing particles: preliminary analysis

The Ir-series PGEs—termed the IPGEs and containing Ir, Ru, or Os,—are a subset of the wider PGE system that behave in a compatible manner, fractionating into crystal phases and readily precipitating from silicate melts, making PGE-bearing crystals a common minor phase in silicate melts in general^21^. Thus the rheological response of silicate melts containing PGE-bearing particles to shear stresses is key to understanding the transport behaviour of mantle-derived melts and their resultant ore deposits^22,23^. Silicate melts that suspend PGE phases are also common in the nuclear industry because the so-called “high-level liquid wastes” (HLLWs) produced by fission^24^ contain PGEs, which when mixed with borosilicate glass in waste vitrification processes, can precipitate to form needle-like RuO_2_ crystals^25^. During vitrification of these wastes, PGE-bearing particles may be found remnant in suspension in the borosilicate glass melt that is produced because they have a low solubility in silicate melts^25^. In magmatic systems, PGE-bearing silicate melts also precipitate PGE-bearing phases, such as alloys, arsenides, and sulfides as crystals^26^. Thus, in both natural and industrial systems, a common feature of PGE-bearing silicate melts is the formation of a suspended fraction of PGE-bearing crystals. In all cases, it is important to understand the rheological impact of these phases, especially given existing evidence that even a low volume fraction of such particles can induce a large and anomalous viscosity change relative to the silicate melt phase alone^15–17^.

**Figure 1:**
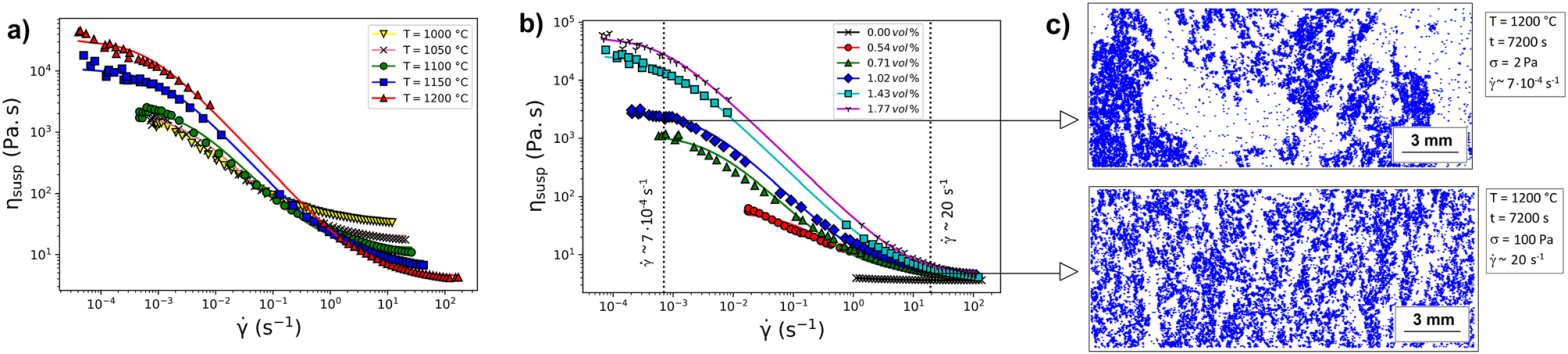
The viscosity of silicate melts suspending PGE-bearing particles as a function of shear strain rate: (**a**) the effect of temperature (Hanotin et al.^17^), (**b**) the effect of PGE-bearing crystal volume percent (Puig et al.^15^), and (**c**) the microstructure of suspensions under two different shear conditions labelled, in which the PGE-bearing particles are displayed in blue and the silicate melt in white (Machado et al.^16^).

Here, we collated experimental data for the viscosity of PGE-bearing melts from Puig et al., Hanotin et al., and Machado et al.^15–17^. Those authors investigated silicate melts containing inclusion of RuO_2_ and Pd-Te alloys. Tellurium does not belong to PGEs, but it forms an alloy with Palladium which is also suspended in the glassy matrix^27^. Figure 1 shows the collated experimental data from the aforementioned references. They were obtained by image analysis (*WebPlotDigitizer*^©^ v4.5) of the published figures from the original articles^28^. Figure 1 also presents the microstructure at low and high shear limits obtained through image analysis of scanning electron microscopy (SEM) images of solidified samples of silicate melts containing PGE-bearing particles^16^.

Suspensions of PGE-bearing particles in silicate melts have been described in previous work via the widely used rheological Cross model^17^. It is a constitutive model used to estimate the apparent shear viscosity of a non-Newtonian fluid between two end-members as a function of shear strain rate. In the case where the non-Newtonian fluid in question is a suspension of particles in a liquid, then this model predicts the suspension viscosity $\upeta_{{{\text{susp}}}}$ as:^29^

1 $$\upeta_{{{\text{susp}}}} = \upeta_{\infty } + \frac{{\upeta_{0} - \upeta_{\infty } }}{{1 + \left( {\dot{\gamma }/\dot{\gamma }_{c} } \right)^{n} }}.$$

where $\upeta_{0}$ and $\upeta_{\infty }$ are the values of $\upeta_{{{\text{susp}}}}$ at the limiting low and high shear strain rates $\dot{\gamma }$, respectively. The parameter $\dot{\gamma }_{c}$ adjusts for the position of the transition between $\upeta_{{{\text{susp}}}} \to \upeta_{0}$ at $\dot{\gamma } \ll \dot{\gamma }_{c}$ and $\upeta_{{{\text{susp}}}} \to \upeta_{\infty }$ at $\dot{\gamma } \gg \dot{\gamma }_{c}$, whereas $n$ controls the shape of this smooth transition. Previous work has suggested that the experimental data for silicate melts containing PGE-bearing particles are well-described by $n = 1$ (termed a simplified Cross fluid model)^17^. In Fig. 1, we confirm that the collated data are well captured by Eq. (1) using $n = 1$, while allowing $\upeta_{0}$, $\upeta_{\infty } ,$ and $\dot{\gamma }_{c}$ to act as free parameters that can be adjusted empirically for each dataset (discussed later).

A possible interpretation of the cross-over between the low ($\upeta_{{{\text{susp}}}} \to \upeta_{0}$ at $\dot{\gamma } \ll \dot{\gamma }_{c}$) and the high ($\upeta_{{{\text{susp}}}} \to \upeta_{\infty }$ at $\dot{\gamma } \gg \dot{\gamma }_{c} )$ shear rate regimes is associated with the observed propensity for these crystal types to form aggregates made up of chains and clumps of particles at low shear rates and above a certain minimum particle fraction^15–17^. Phenomenologically, these aggregates appear to increase drastically the viscosity of the suspension. Conversely, at high-shear regimes, the material is composed of distributed individual particles and behaves as a classical suspension. The cause of these shear rate-mediated differences is of central interest here.

In the data used here, the PGE-bearing inclusions make up a relatively low volume fraction of the total suspension, of which ~ 67 vol.% are acicular RuO_2_ crystals and ~ 33 vol.% are Pd-Te spheres. These particles have sizes in the range of 5–10 µm (RuO_2_ needle length) and 0.5–2.5 µm (Pd-Te radius), respectively^16^. RuO_2_ needles have aspect ratio R varying in the range 5 < R < 20 and the Pd-Te spheres have R = 1^15^. Following a similar laboratory procedure as both Puig et al^15^. and Hanotin et al.^17^, Machado et al.^16^ applied a pre-shearing step at applied shear stress σ = 200 Pa for t = 300 s in order to break up the PGE aggregates. The mean radius of the suspended individual particles (${\text{a }}$ = 3.92 ± 0.35 µm) and was found via image analysis of Fig. 1 in Machado et al.^16^. The suspending phase is a borosilicate melt (for full composition we refer the reader to the original works) and the temperature dependence of the liquid viscosity η follows a Vogel-Fulcher-Tammann law (VFT) of the form $\upeta = {\text{A}}\exp \left[ {{\text{B/}}\left( {{\text{T}} - {\text{T}}_{0} } \right)} \right]$, with A = 0.007 Pa.s, B = 5251 K and T_0_ = 675 K.

## Comparison between silicate suspensions with different particle types

**Figure 2:**
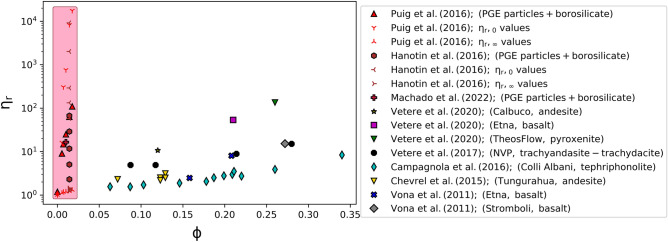
Relative viscosity values obtained at $\dot{\gamma } = 0.1 \;{\text{s}}^{ - 1}$ (filled symbols with an outline) and limiting relative viscosity values $\upeta_{{{\text{r}},0}}$ and $\upeta_{{{\text{r}},\infty }}$ (skeletal symbols) as a function of crystal volume fraction $\phi$ for different crystal-bearing silicate melts across a wide range of crystal types and melt compositions.

Rheological data of melts containing PGE-bearing particles were extracted from Puig et al., Hanotin et al., and Machado et al.^15–17^. As a first step, we compare these data with results from published experimental work on silicate melts suspending other crystal types across a range of crystal volume fractions^10–14^. In order to perform this comparison we define the relative viscosity as $\upeta_{{\text{r}}} = \upeta_{{{\text{susp}}}} {/}\upeta$. It is clear from Fig. 1, that this normalization by the melt viscosity does not change the form of the shear rate dependence, and therefore Eq. (1) becomes

2 $$\upeta_{{\text{r}}} = \upeta_{{{\text{r}},\infty }} + \frac{{\upeta_{{{\text{r}},0}} - \upeta_{{{\text{r}},\infty }} }}{{1 + \left( {\dot{\gamma }/\dot{\gamma }_{c} } \right)^{n} }},$$

where with reference to Eq. (1), $\upeta_{{{\text{r}},0}} = \upeta_{0} /\upeta$, $\upeta_{{{\text{r}},\infty }} = \upeta_{\infty } /\upeta$, and $n$ remains 1. Fitting Eq. (2) to the collated data, we can extract the limiting viscosity values $\upeta_{{{\text{r}},0}}$ and $\upeta_{{{\text{r}},\infty }}$ for comparison with other data. However, rheological data for silicate melts containing other crystal types have not been analysed using Eq. (2), and therefore do not have an associated $\upeta_{{{\text{r}},0}}$ and $\upeta_{{{\text{r}},\infty }}$ pair. For this reason, we additionally extract a viscosity at $\dot{\gamma } = 0.1 \;{\text{s}}^{ - 1}$. The viscosity values were obtained either by taking measurements executed at $\dot{\gamma }$ = 0.1 s^−1^ or by interpolating experimental data at this specific shear rate value. No data have been obtained by extrapolation. The aspect ratios of the compared crystals vary from R ~ 2 to ~ 14^10–14^. Figure 2 assembles different η_r_ values obtained at $\dot{\gamma } = 0.1 \;{\text{s}}^{ - 1}$ as well as the limiting values $\upeta_{{{\text{r}},0}}$ and $\upeta_{{{\text{r}},\infty }}$ obtained from the fitting procedures (table S1)^15,17^ as a function of crystal volume fraction. Hanotin et al.^17^ do not give the fit parameters and so, for this study, we fitted their data, while Puig et al.^15^ fitting values were collated directly from the reference. The pink shaded area in Fig. 2 corresponds to the region in which the relative viscosity of the system suspending PGE-bearing particles can vary according to the experiments of Puig et al.^15^ and Hanotin et al.^17^ Here, the collated data are mainly focussed on systems relevant to nuclear waste vitrification and volcanic eruptions or magmatism. More information on this comparison can be found in table S1, table S2, and in the references themselves^10–17^.

Figure 2 confirms that, for $\dot{\gamma } = 0.1\;{\text{s}}^{ - 1}$, suspensions of PGE-bearing particles present viscosities much higher than their crystal-bearing magmatic counterparts. These PGE-bearing suspensions present, at small particle volume fractions ($\phi$ ≲ 0.02), roughly the same or higher relative viscosity than magmas containing $0.2 { \lesssim }\phi { \lesssim }0.35$. It is worth noting here that the PGEs are sparingly soluble in silicate melts^25,30^, while the silicate crystals are formed due to crystallisation from the major elements of the parental melt. Thus, the chemical nature of PGE-bearing particles is different from that of the suspending silicate liquid, whereas for magmatic silicate crystals the chemical contrast to the melt phase is lower. Figure 2 also shows that the limiting values of $\upeta_{{{\text{r}},0}}$ and $\upeta_{{{\text{r}},\infty }}$ form two discrete trends that are very different in terms of $\upeta_{{\text{r}}} \left( \phi \right)$. The latter is qualitatively more consistent with the silicate suspension data, whereas the former is close to the data assessed at $\dot{\gamma } = 0.1 \;{\text{s}}^{ - 1}$. The values of relative viscosity for the same particle fraction different in detail as the experiments were performed at different temperatures whereby the aggregation and rheology behaviour of PGE-bearing particles are both temperature-dependent.

## Comparison with rheological models

Suspension rheology models are compiled in this section with the purpose of comparison with the collated rheological data for suspensions of PGE-bearing particles. Puig et al.^15^ compared viscosity data for varying particle content of PGE-bearing melts with some selected models. However, they exclusively dealt with the high shear rate conditions ($\upeta_{\infty }$ values), in which the inclusions occur in small clusters or individual particles and the bulk fluid behaves as a classical suspension. The low shear rate viscosity $\upeta_{0}$ of these suspensions also warrants explanation. The rheology of a particle suspension is a complex function of the microstructural properties. The parameters commonly used for the characterization of these solid-bearing silicate melts include the nature of the bulk fluid, volume fraction of phases, and crystal shapes and sizes^4,8^. Another factor that has not been invoked for silicate suspensions, but is commonly invoked in colloids, is interparticle forces.

Einstein^31^ developed a model to predict the viscosity of liquids containing solid spherical monodisperse particles in the dilute regimes:

3 $$\upeta_{{\text{r}}} = 1 + {\text{B}}\phi ,$$

where B is the intrinsic viscosity or Einstein coefficient, commonly assumed to be equal to 2.5. This model predicts that viscosity grows linearly with particle volume fraction. Rutgers^32,33^ suggested that the Einstein^31^ model is valid for $\phi$ ≲ 0.02. To expand the range of applicability to higher particle volume fractions, Roscoe^34^ found:

4 $$\upeta_{{\text{r}}} = \left( {1 - 1.35\phi } \right)^{ - 2.5} ,$$

commonly referred to as the Einstein-Roscoe (ER) equation. Krieger and Dougherty^35^ proposed:

5 $$\upeta_{{\text{r}}} = \left( {1 - \frac{\phi }{{\phi_{{\text{m}}} }}} \right)^{{ - {\text{B}}\phi_{{\text{m}}} }} ,$$

where the B is the intrinsic viscosity (cf. Equation 3) and $\phi_{m}$ is the maximum packing fraction. $\phi_{m}$ is the volume fraction beyond which there is no remaining space for translation movement and particle accommodation. In rheological terms, $\phi_{m}$ is defined as the volume fraction at which particles can no longer flow and consequently the suspension becomes ‘jammed’^8^. Thus, at this particle volume fraction, Eq. (5) predicts that the suspension viscosity tends towards infinity^6^.

Maron & Pierce^36^ derived an equation with the same functional form as the Krieger and Dougherty^35^ equation (Eq. 5) but with ${\text{B}}\phi_{m}$ = 2, suggesting a solution with just one adjustable parameter, $\phi_{{\text{m}}}$.

6 $$\upeta_{{\text{r}}} = \left( {1 - \frac{\phi }{{\phi_{{\text{m}}} }}} \right)^{ - 2} .$$

The Krieger and Dougherty^35^ equation (Eq. 5), and the Maron & Pierce^36^ equation (Eq. 6) require constraint of ${\text{B}}$ and $\phi_{{\text{m}}}$, and just $\phi_{{\text{m}}}$, respectively. These are parameters that may encode the properties of the particles present in terms of their shape (captured by the shape factor ${\text{R}}$). There exist various approaches to render ${\text{B}}$ a function of ${\text{R}}$. Here we focus on two approaches commonly used for silicate systems: Ishibashi and Sato^37^ (Eq. 7a), and Mueller et al.^8^ (Eq. 7b) give ${\text{B}}\left( {\text{R}} \right)$ solutions.

7a $${\text{B}} = 1.642 + 0.512{\text{R}},$$

7b $${\text{B}} = 3.02 + 0.321{\text{R}}.$$

Hence, using the aforementioned relation ${\text{B}}\phi_{{\text{m}}}$ = 2 (Mueller et al.^8^ approach), Eq. (7) leads to expressions for the $\phi_{{\text{m}}}$ as follows:

8a $$\upphi_{{\text{m}}} = \frac{2}{{1.642R + 0.512{\text{R}}}}.$$

8b $$\upphi_{{\text{m}}} = \frac{2}{{0.302 + 3.21{\text{R}}}}.$$

For the 5 < R < 20 of the RuO_2_ crystals and R = 1 of the Pd-Te particles considered here, Eq. (8) predict average values of maximum packing fractions as $\phi_{{\text{m}}} = 0.473$ and $\phi_{{\text{m}}} = 0.388$, for Ishibashi and Sato^37^ and Mueller et al.^8^, respectively. By contrast, Puig et al.^15^ used an approximate method to fit for $\phi_{{\text{m}}}$ using the $\upeta_{{{\text{r}},\infty }}$ data and found $\phi_{{\text{m}}} = 0.119$, while Hanotin et al.^17^ fit a functional form equivalent to Eq. (6) to the $\upeta_{{{\text{r}},\infty }}$ data and found $\phi_{{\text{m}}} = 0.122$.

**Figure 3:**
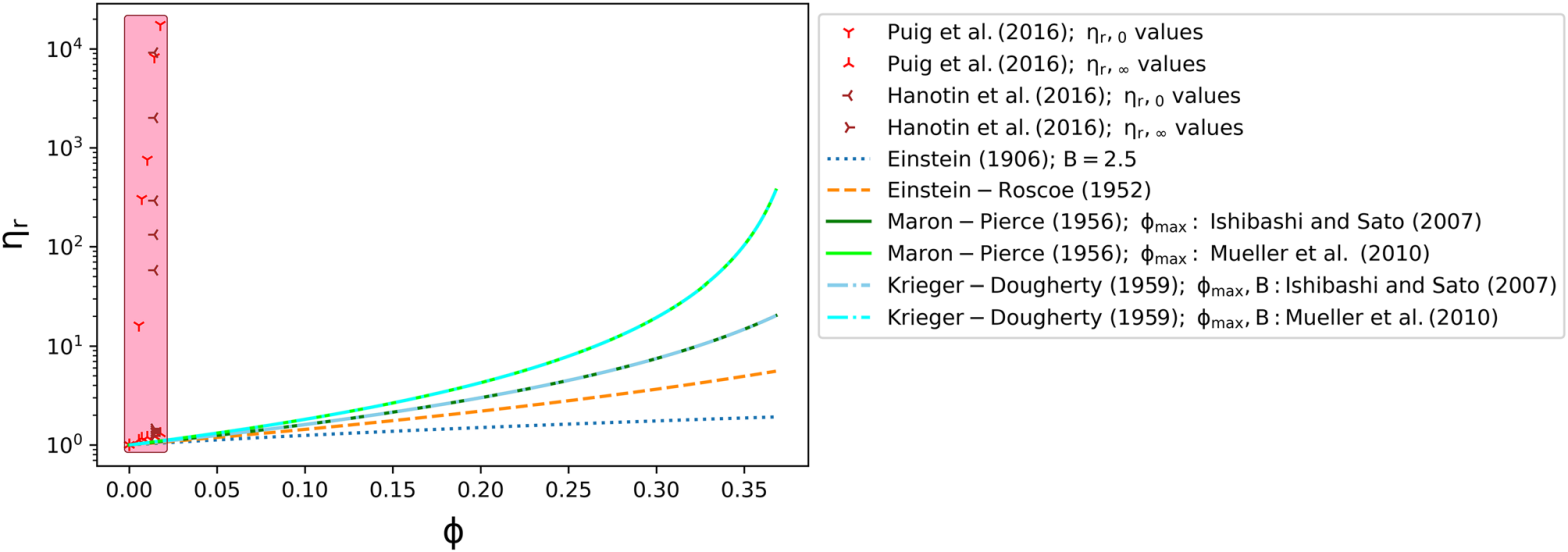
Relative viscosity as function of crystallinity for the collated literature data on PGE-bearing melts obtained at $\upeta_{{{\text{r}},0}}$ and $\upeta_{{{\text{r}},\infty }}$ compared to different rheological models. The considered parameters are labelled for the models.

In Fig. 3, we test the rheological models given here against the data for silicate melts suspending PGE-bearing particles. We show the result of solving Eqs. (3)–(6) assuming the calculated $\phi_{{\text{m}}}$ values ($\phi_{{\text{m}}} = 0.473$, $\phi_{{\text{m}}} = 0.388$). We find that in all cases, the data for $\upeta_{{{\text{r}},\infty }}$ are relatively well approximated by the models. However, the data for low shear rate regimes $\upeta_{{{\text{r}},0}}$ are not predicted by these models. Figure 3 confirms that the aforementioned classical rheological models for suspended particles in liquids do not describe well the rheological behaviour of silicate melts suspending PGE-bearing particles at relatively low shear rates.

We speculate however that at high shear rates where $\upeta_{{\text{r}}} = \upeta_{{{\text{r}},\infty }}$, these suspensions of PGE-bearing particles behave as classical suspensions and therefore either of Eqs. (5) and (6) could be used to estimate the experimental values obtained therein.

**Figure 4:**
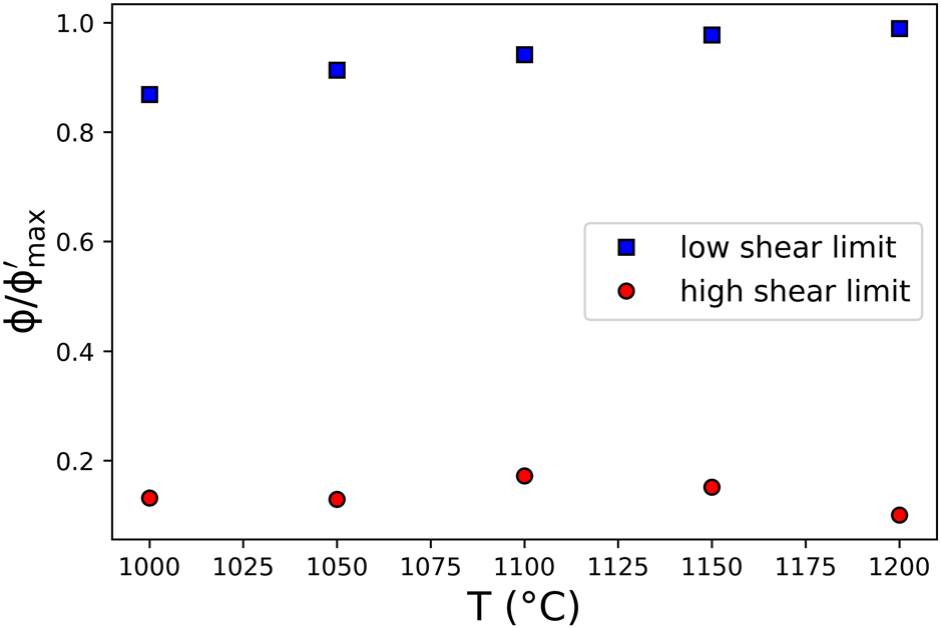
The effective particle cluster volume fraction in the low shear rate regime (blue squares) along with the effective particle cluster volume fraction in the high shear rate regime (red circles). The maximum packing fraction for low and high shear scenarios are displayed as dotted lines of their respectively colours.

In order to understand the behaviour at low shear rates; where $\upeta_{{\text{r}}} = \upeta_{{{\text{r}},0}}$, we appeal to abundant evidence^2,15–17,38^ that PGE-bearing particles aggregate to form clusters (see also Fig. 1c herein and Fig. 6b presented in Hanotin et al.)^17^. Clusters of needle-like RuO_2_ crystals and Pd-Te spherical particles are likely to be jammed at relatively low local volume fractions (c.f. Equations 8), and could therefore trap so-called ‘dead melt’ between them, where ‘dead melt’ refers to a fraction of the liquid phase that cannot contribute to the hydrodynamics and is stationary relative to the enclosing crystals even under shear. If the formation of fractions of ‘dead melt’ is the consequence of aggregation of the PGE-bearing particles, then the ‘dead melt’ effectively is as much an obstacle to the ‘active melt’ as the crystals themselves are. This has the effect of increasing the volume fraction of unmoving phase—termed $\phi^{\prime }$ in the system. At high shear rates $\phi^{\prime } \approx \phi$ because the crystals are the only unmoving phase, whereas at relatively low shear rates, $\phi^{\prime } > \phi$ due to aggregation and trapping fluid. To illustrate this, we estimate $\phi^{\prime } /\phi_{m}$ at two situations: a) $\dot{\gamma } \ll \dot{\gamma }_{c}$ and b) $\dot{\gamma } \gg \dot{\gamma }_{c}$. We select Eq. (6) for simplicity, as it only requires a single unknown parameter $\phi_{{\text{m}}}$. Because $\upeta_{{\text{r}}}$ is a measured quantity, for each measurement, we can rearrange Eq. (6) to find the equivalent normalised $\phi /\phi_{{\text{m}}}$, which is $\phi^{\prime } /\phi_{{\text{m}}}$, that would be required to explain the data: $\phi^{\prime } /\phi_{{\text{m}}} = 1 - \upeta_{{\text{r}}}^{ - 1/2}$. Using the Hanotin et al.^17^ data, in Fig. 4 we demonstrate that this value $\phi^{\prime }$—the aggregated particle cluster volume fraction—appears to be dependent on the temperature for low shear regimes, similarly to the aggregation tendency. Moreover, for low shear scenarios, $\phi^{\prime }$ values are close to the maximum packing fraction. On the other hand, for high shear scenarios, these values are much lower than the maximum packing fraction and close to the real particle volume fraction. Thus, almost no ‘dead melt’ exists at high shear stresses. This observation is used in “Universal scaling of PGE-bearing melt viscosity” section to underpin our interpretation and scaling approach for these results.

## Universal scaling of PGE-bearing melt viscosity

The analysis given in Fig. 4 suggests that a temperature-dependence of crystal aggregation is a plausible mechanism in the low shear rate regime. Temperature-dependent particle motion is predicted by theory underpinning Brownian suspensions. For suspensions of hard particles under Brownian motion, the characteristic timescale of particle motion is λ_Br_, which corresponds to the diffusion time of a particle over a distance equivalent to its radius^18,39,40^ and is

9 $$\uplambda_{{{\text{Br}}}} = \frac{{6\uppi \upeta {\text{a}}^{3} }}{{{\text{kT}}}},$$

where ${\text{a}}$ is the suspended particle radius, $k$ is the Boltzmann constant, and ${\text{T}}$ is the absolute temperature. Similarly, there is a timescale λ_hy_ associated with the hydrodynamics under shear deformation^18,39,40^

10 $$\uplambda_{{{\text{hy}}}} \sim \dot{\gamma }^{ - 1} .$$

Considering interactive particles suspended in a liquid, a characteristic time associated to the potential interaction of particles ${\text{U}}$ can be also defined as^39^:

11 $$\uplambda_{{\text{int}}} = \frac{{6\uppi \upeta {\text{a}}^{3} }}{{\text{U}}}.$$

Berli and Quemada^18^ defined an expression for the characteristic time of particles being rearranged over Brownian motion and interparticle forces λ_Br-int_, which can be written as:

12 $$\uplambda_{{{\text{Br}} - {\text{int}} }} = \frac{{6\uppi \upeta {\text{a}}^{3} }}{{{\text{kT}} + {\text{U}}}}.$$

Considering the characteristic timescales $\uplambda_{{{\text{Br}}}}$, $\uplambda_{{{\text{Br}} - {\text{int}} }} ,$ and $\uplambda_{{{\text{hy}}}}$, we can define two Peclet numbers: ${\text{Pe}}_{{{\text{non}} - {\text{int}}}}$ and ${\text{Pe}}_{{{\text{int}}}}$

13a $${\text{Pe}}_{{{\text{non}} - {\text{int}}}} = \frac{{\uplambda_{{{\text{Br}}}} }}{{\uplambda_{{{\text{hy}}}} }} = \frac{{6\uppi \upeta {\text{a}}^{3} }}{{{\text{kT}}}}\dot{\gamma },$$

13a $${\text{Pe}}_{{\text{int}}} = \frac{{\uplambda_{{{\text{Br}} - {\text{int}} }} }}{{\uplambda_{{{\text{hy}}}} }} = \frac{{6\uppi \upeta {\text{a}}^{3} }}{{{\text{kT}} + {\text{U}}}}\dot{\gamma }.$$

where at low Pe (either ${\text{Pe}}_{{{\text{non}} - {\text{int}}}}$ or ${\text{Pe}}_{{{\text{int}}}}$), the diffusional forces dominate over hydrodynamic forces and one might expect the microstructural properties of colloids to tend towards an isotropic equilibrium. On the other hand, at large Pe, shear forces locally disrupt microstructural equilibrium.

**Figure 5:**
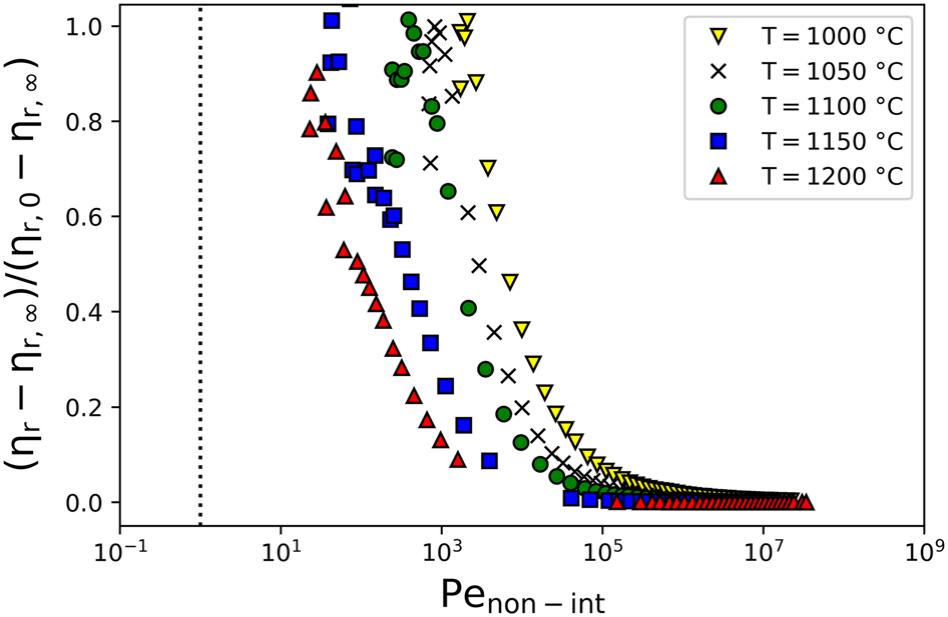
Normalised relative viscosity data from Hanotin et al.^17^ against Pe number for non-interactive particles.

To scale the rheological data from Hanotin et al.^17^, we provide a two-step approach. First, we use the fits to Eq. (2) to scale the viscosity as $y = \left( {\eta_{r} - \eta_{r,\infty } } \right)/\left( {\eta_{r,0} - \eta_{r,\infty } } \right)$, such that Eq. (2) becomes $y = 1/\left( {1 + \gamma \dot{/}\dot{\gamma }_{c} } \right)$, assuming $n = 1$, as discussed earlier. Figure 5 shows the rescaled viscosity data from Hanotin et al.^17^ against ${\text{Pe}}_{{{\text{non}} - {\text{int}}}}$ and that does not present a universal behaviour. The transition between the high- and low-viscosity plateaus happens at a critical Peclet number Pe_c_ corresponding to the best-fit $\dot{\gamma }_{c}$, which appears to be temperature dependent.

**Figure 6:**
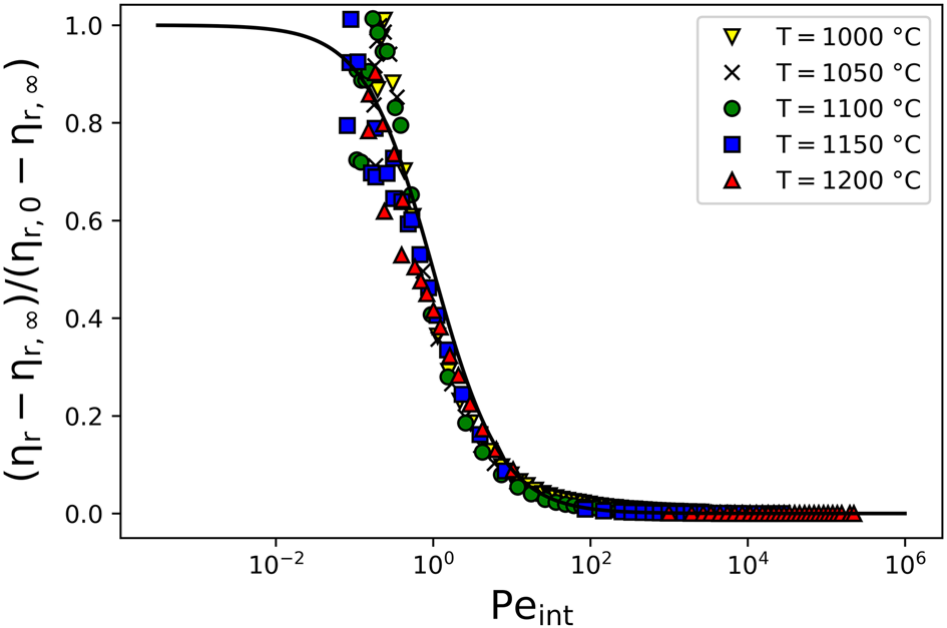
Normalised relative viscosity from Hanotin et al.^17^ against Pe number for interactive particles.

Given our interpretation that the formation of the particle aggregate clusters at low shear rates is associated with thermally-driven, particle–particle interactions, we propose that ${\text{U}} > 0$ for melt suspensions of PGE-bearing particles (c.f. Equation 13b). In order to account for U, we first refer to Foss & Brady^41^, who studied inert particles in suspension, and find that across a wide range of $\phi$, ${\text{Pe}}_{{{\text{non}} - {\text{int}}}} \approx 1$ is the critical Peclet number for a transition from high to low relative viscosity with increasing shear rate. This is backed by Quemada^39^, who also invoked the same value for the transition. Therefore, we define a solution to the rescaled form of Eq. (2) such that $\dot{\gamma }_{c}$ coincides with ${\text{Pe}} = 1$ by $y = 1/\left( {1 + {\text{Pe}}_{{{\text{int}}}} } \right)$, and use this to fit for the value of U for each temperature that would be required to adjust the data to this curve (Fig. 6).

Therefore, by observing the collapsed data plotted in Fig. 6, it is important to recognise that for this temperature range (1000–1200 °C), the mechanisms dictating particle aggregation in this borosilicate melt are self-similar. We could get the observed universal behaviour by invoking hydrodynamic, Brownian, and interparticle forces. As expected the transition between the high and low-viscosity plateaus takes place at ${\text{Pe}}_{{{\text{int}}}} \approx 1$. It is interesting to observe here that the low-shear viscosity plateau mentioned by the Hanotin et al.^17^ is not as pronounced as in the non-scaled figure.

## Discussion

Using experimental data for the rheology of melts suspending PGE-bearing particles, we have demonstrated that there is a low shear deformation rate viscosity for these suspensions that is far in excess of the equivalent for suspensions of silicate crystals. Moreover, no rheological model predicts this behaviour. It has been proposed that this low shear rate viscosity is related to the aggregation tendency of PGE-bearing particles when suspended in silicate melts^17^. By considering hydrodynamic, Brownian, and interparticle forces, a universal behaviour of the rheological data of melts suspending PGE-bearing particles could be obtained. In this section, we discuss the presence of such interparticle forces and we also present other evidence supporting the interpretation that they should be present in these types of system.

Some work on melts suspending PGE-bearing particles have invoked the presence of hydrodynamic, Brownian, and interparticle forces^15–17^. However, the novel approach here has been to rationalise the influence of these forces on particle aggregation at high-temperature in silicate liquids. Our results suggest two important things: (1) that at high relative to low temperatures, $\eta_{{{\text{r}},0}}$ is relatively high, implying a higher $\phi^{\prime }$ (Fig. 4); and (2) that at relatively high temperatures, $\dot{\gamma }_{c}$ and ${\text{U}}$ are low. In the context of Brownian and interparticle forces, this can be understood in terms of the thermal motion involved. At high relative to low temperatures, Brownian motion is more vigorous, and considering the results at low shear rates (Fig. 1a), it seems reasonable that cluster formation would be more efficient at higher temperatures. Moreover, it is also plausible that these clusters, formed at high relative temperatures, would be more disordered than at lower relative temperatures for which that Brownian vigour is lower. Similarly, the shear rates required to disrupt clusters, which is our interpretation of the physical meaning of $\dot{\gamma }_{c}$, is relatively low at higher temperatures, such that ${\text{U }}$ is also low. This can be rationalised by the low relative force of particle–particle interactions maintaining the clusters at higher relative temperatures.

In addition to the conceptual arguments posed above, we consider the physico-chemistry of the system in the context of colloids to explain particle assembly. This union can be either called agglomeration or aggregation. While the former refers to an irreversible process, the latter refers to a reversible one. The so-called DLVO (Derjaguin, Landau, Verwey, Overbeek) theory^42,43^ describes the assemblage of dispersed particles and can be simply explained by the interplay between the attractive van der Waals and the repulsive electrostatic double-layer (EDL) forces. Van der Waals energy between two identical particles in a medium is always negative (attractive) and may thus promote particle union^44^. On the other hand, in aqueous colloidal suspensions for example, particles are surrounded by electrolyte ions^44^. As two particles approach each other, overlapping of the mentioned layers leads to a repulsive force, which in turn prevents particle aggregation. This creates a repulsive force which is called electrostatic double layer. The electrochemistry of molten oxide-glasses generally correlates with that of aqueous solutions^45^. This parallel is also made via the investigation of glass basicity^46^. Silicate melt network formers (Si, B, Al) have the role of creating the spatial arrangement in which the network modifiers (Na, Ca) have partial freedom of movement. In the presence of suspended charge-bearing crystals, these ions surround them, diminishing the EDL effect^44^. Van der Waals potential is not a direct function of ion concentration in the medium, while the EDL contribution decreases for increasing the ion concentration. Therefore, in this context, the van der Waals attractive force may prevail and the system undergoes aggregation more easily if ions segregate around the suspended inclusions. Furthermore, the intensities of the DLVO forces are functions of the suspended particle size, in which Genovese et al.^19^ stated to be relevant for particles up to ~ 10 µm.

Nuernberg et al.^38^ show an anomaly in the electrical behaviour of RuO_2_-bearing glasses and melts using the above-mentioned rearrangement of counterions (Na^+^, Ca^+2^) around RuO_2_ crystals. They measured the electrical conductivity of RuO_2_-containing melts employing impedance spectroscopy. A decrease of ionic conductivity of these composite melts is observed when submitted to cyclic electrical conductivity measurements. They raise a hypothesis that the drop of the ionic conductivity could be linked to a regrouping of Na^+^ and Ca^+2^ on the surface of RuO_2_ particles. Indeed, if these mobile species are trapped on RuO_2_ surface, there will be less ionic species available to the charge conduction process, and consequently ionic conductivity would drop. Cabaret et al.^47^ have detected, through XANES spectroscopy, that the incorporation of poorly soluble noble metals in borosilicate melts drives a rearrangement of mobile atoms within the vitreous network. They observe, that in the presence of these noble particles, the glassy network becomes more polymerised due to the reorganisation of network modifiers around RuO_2_ particles.

Besides the presented evidence of possible rearrangement of ions around RuO_2_ crystals in silicate melts, supporting the idea that interparticle forces may be present, it has also been observed that a nano-scaled equilibrium distance exists between intergranular films in polycrystalline ceramics^48,49^. Those studies confirm that under certain assumptions, the mentioned intergranular equilibrium distance is in mathematical and physical agreement with principles of the balance between the attractive van der Waals and repulsive EDL forces^49^. More recently, evidence has emerged that thick-film resistors also exhibit an equilibrium nanometric distance between particles in a glassy matrix^50^. This composite material is composed of ultrafine particles of Pb_2_Ru_2_O_7_ in a highly modified silicate glass matrix, similar to the materials of interest in this study.

Apart from these evidences, it is also important to mentioned that in Pereira et al.^1^, we measured the contact angle between air bubbles and RuO_2_ crystals immersed in a borosilicate melt. This contact angle is one of the largest found in literature along with magnetite^3,51^. Thus, here we also invoke the possibility of Marangoni effect helping the aggregation of PGE-bearing particles in this type of silicate melt, but a detailed study rationalising its influence should be carried out before any conclusion.

Different lines of evidence point to the likelihood that interparticle forces should be present in silicate melts suspending PGE-bearing particles inviting in turn the application of the DLVO theory. Having explored conceptual and colloidal arguments we are able to justify the introduction of the U term to account for the interparticle potential energy that governs the shear-thinning effect in the suspensions studied herein. The scaling law presented, which considers hydrodynamic, Brownian, and interparticle forces can be used to describe the scaling behaviour for PGE-bearing silicate melt suspensions.

## Conclusion

In this work, to understand the rheological behaviour of PGE-bearing silicate melts, we collated experimental data from the literature. Despite of the low particle volume fraction ($\phi$ ~ 0.02) of PGE-bearing suspensions, a disproportional increase of the suspension viscosities was observed, especially at low Pe number. Based on comparison with other rheological data, as well as with rheological models, we demonstrate that PGE-bearing particles increase the suspension viscosity much more than silicate crystals due to aggregation in clusters. Furthermore, we scaled the relative viscosity of PGE-bearing melts using a) a Pe number for non-interactive particles and b) a Pe number for interactive particles. The universal scaling law for the rheological behaviour of PGE-bearing silicate melts could be obtained for the latter case. Finally, evidence was presented to explain why one should consider interactive particles in the case of PGE-bearing particles in silicate melts.

## Supplementary Information

Supplementary Information 1.

Supplementary Information 2.
