# Supplementary material for: Interparticle and Brownian forces controlling particle aggregation and rheology of silicate melts containing platinum-group element particles
Source: Sci Rep. 2022 Jun 2;12:9226. doi: 10.1038/s41598-022-12948-1 (PMC9163177; doi:10.1038/s41598-022-12948-1)
Supplement: Supplementary file 1 — Supplementary Information 1. [file 41598_2022_12948_MOESM1_ESM.docx]

Table S1: Fitting parameters of Hanotin et al.^17^ data and fit values displayed in Puig et al. for the suspensions of PGE-bearing particles in silicate melts.

| Reference | T (°C) | vol.% | γ_c_ (s^-1^) | η_0_ (Pa.s) | η_∞_ (Pa.s) |
| --- | --- | --- | --- | --- | --- |
| Puig et al. (2016) | 1200 | 0 | 0 | 3.60e00 | 3.60e00 |
| Puig et al. (2016) | 1200 | 0.544 | 1.10e-01 | 5.80e01 | 4.00e00 |
| Puig et al. (2016) | 1200 | 0.714 | 5.80e-03 | 1.10e03 | 4.20e00 |
| Puig et al. (2016) | 1200 | 1.020 | 3.30e-03 | 2.72e03 | 4.30e00 |
| Puig et al. (2016) | 1200 | 1.428 | 7.20e-04 | 2.97e04 | 4.40e00 |
| Puig et al. (2016) | 1200 | 1.768 | 5.70e-04 | 6.30e04 | 4.70e00 |
| Hanotin et al. (2016) | 1000 | 1.428 | 2.96e-03 | 1.67e03 | 3.83e01 |
| Hanotin et al. (2016) | 1050 | 1.428 | 3.08e-03 | 2.02e03 | 2.00e01 |
| Hanotin et al. (2016) | 1100 | 1.428 | 3.18 e-03 | 2.56e03 | 1.28e01 |
| Hanotin et al. (2016) | 1500 | 1.428 | 1.18e-03 | 1.09e04 | 7.55e00 |
| Hanotin et al. (2016) | 1200 | 1.428 | 6.35e-04 | 3.28e04 | 4.43e00 |
