# Supplementary material for: Interparticle and Brownian forces controlling particle aggregation and rheology of silicate melts containing platinum-group element particles
Source: Sci Rep. 2022 Jun 2;12:9226. doi: 10.1038/s41598-022-12948-1 (PMC9163177; doi:10.1038/s41598-022-12948-1)
Supplement: Supplementary file 2 — Supplementary Information 2. [file 41598_2022_12948_MOESM2_ESM.docx]

Table S2: Crystal-bearing melt system information.

| Reference | Material | Particles | Shear rate (s^-1^) | Crystal size range |
| --- | --- | --- | --- | --- |
| Puig et al. (2016) | PGE + borosilicate melt (Nuclear waste glass melt) | RuO_2_ | 0.1 | μm |
|  |  | Pd-Te |  |  |
| Hanotin et al. (2016) | PGE + borosilicate melt  (Nuclear waste glass melt) | RuO_2_ | 0.1 | μm |
|  |  | Pd-Te |  |  |
| Machado et al. (2022) | PGE + borosilicate melt  (Nuclear waste glass melt) | RuO_2_ | 0.1 | μm |
|  |  | Pd-Te |  |  |
| Vetere et Holtz (2020) | Andesite (Calbuco) | Plagioclases^*^ | 0.1 | μm |
|  |  | Fe-Ti oxides |  |  |
| Vetere et Holtz (2020) | Basalt (Etna) | Plagioclases^*^ | 0.1 | μm |
|  |  | Fe-Ti oxides |  |  |
| Vetere et Holtz (2020) | Pyroxenite (Theo’s flow) | Clinopyroxene | 0.1 | μm |
| Vetere et al. (2017) | Trachyandesite - Trachydasite (Mercury NVP) | Olivine | 0.1 | μm |
|  |  | Pyroxene |  |  |
| Campagnola et al. (2016) | Tephriphonolite (Colli Albani) | Leucite^*^  Plagioclase | 0.1 | μm |
| Chevrel et al. (2015) | Andesite (Tungurahua) | Plagioclase^*^ | 0.1 | μm |
|  |  | Oxides |  |  |
| Vona et al. (2011) | Basalt (Etna) | Plagioclase^*^ | 0.1 | μm |
|  |  | Spinel |  |  |
|  |  | Pyroxene |  |  |
| Vona et al. (2011) | Basalt (Stromboli) | Plagioclase^*^ | 0.1 | μm |
|  |  | Spinel |  |  |
|  |  | Pyroxene |  |  |

* main crystalline phase
